# Supplementary material for: Simulating pedigrees ascertained for multiple disease-affected relatives
Source: Source Code Biol Med. 2018 Oct 15;13:2. doi: 10.1186/s13029-018-0069-6 (PMC6190569; doi:10.1186/s13029-018-0069-6)
Supplement: Supplementary file 1 — SimRVPedigree Supplement. This is a pdf file that provides detailed information about the simulation procedure, as well as additional information for the applications discussed in the main text. (PDF 254 kb) [file 13029_2018_69_MOESM1_ESM.pdf]

Supplement to Simulating Pedigrees Ascertained for Multiple Disease-Affected Relatives

Christina Nieuwoudt, Samantha J. Jones, Angela Brooks-Wilson and Jinko Graham

1 Algorithm to Simulate All Life Events Starting at Birth

To simulate all life events for an individual, starting at birth, we implement the following algorithm, which simulates life events until either death or a simulated event exceeds the last year of the study.

- Set y to the individual's year of birth.
- Set y_S to the last year of the study.
- Set $t_{max} = y_S - y$.
- Set $t = 0$. In this context, t represents the individual's age, in years; hence, at birth the individual is 0 years old.
- Determine the individual's risk variant status, x, where $x = 1$ if the individual has the familial risk variant and $x = 0$ otherwise.
- Set δ, the disease status indicator, to 0 to indicate that disease onset has not occurred at birth.
- While $t < t_{max}$:
  - Simulate $w_{o|t,x}$, the waiting time to disease onset conditioned on the current age and rare-variant status[1].
  - Simulate $w_{d|t,\delta}$, the waiting time to death conditioned on the current age and disease status.
  - Simulate $w_{r|t}$, the waiting time to reproduction conditioned on the current age.
  - Set $t' = min\{\ w_{o|t,x},\ w_{d|t,\delta},\ w_{r|t}\ \}$.
  - If $t + t' < t_{max}$ and $t' = w_{o|t,x}$:
    - set $\delta = 1$,
    - store the individual's year of disease onset, $y + t + t'$,
    - and set $t = t + t'$.
  - If $t + t' < t_{max}$ and $t' = w_{d|t,\delta}$:
    - store the individual's year of death, $y + t + t'$,
    - set $t = t_{max}$ to stop the simulation.
  - If $t + t' < t_{max}$ and $t' = w_{r|t}$:
    - create offspring, store offspring's year of birth, $y + t + t'$, simulate the offspring's gender uniformly between male and female, and simulate the offspring's rare-variant status according to Mendel's laws
    - set $t = t + t'$.
  - If $t + t' \geq t_{max}$, set $t = t + t'$ (i.e. stop simulation).

[1]Details for simulating $w_{o|t,x}$, $w_{d|t,\delta}$, and $w_{r|t}$ may be found in the main text in *Methods: Simulating Life Events*.

2 Distribution of Average IBD Probability Among Affected Family Members

We measure familial disease clustering by the average of the pairwise identity by descent (IBD) probabilities among the affected relatives in the pedigree. We denote this measure by by $\mathcal{A}_{IBD}$. To formalize this measure, within a pedigree, we denote the k affected family members by $m_1, m_2, ..., m_k$, and let $p_{i,j}$ denote the probability that m_i and m_j share a variant IBD. Using this criteria, $\mathcal{A}_{IBD}$ may be calculated as

$$\mathcal{A}_{IBD} = \frac{\sum_{i \neq j} p_{i,j}}{\binom{k}{2}}.$$

To investigate the relationship between familial clustering among affected relatives and κ, the relative-risk of disease in genetic cases, we consider three genetic-relative-risk groups: $\kappa = 1$, $\kappa = 10$, and $\kappa = 20$. The simulated study samples are described in the main text in section *Results: Familial Clustering.*

Tables 1 and 2 summarize the conditional distribution of $\mathcal{A}_{IBD}$ in families with two and three disease-affected relatives, respectively, for the three genetic-relative-risk groups considered.

Table 1: Summary of conditional distributions of $\mathcal{A}_{IBD}$ for pedigrees with two disease-affected relatives.

Genetic		P($\mathcal{A}_{IBD} = \alpha$)			
Relative	Sample	α			
Risk, κ	Size	0.0625	0.125	0.25	0.5
$\kappa = 1$	959	0.0073	0.0761	0.2492	0.6674
$\kappa = 10$	827	0.0000	0.0314	0.1475	0.8210
$\kappa = 20$	748	0.0000	0.0120	0.1324	0.8556

Table 2: Summary of conditional distributions of $\mathcal{A}_{IBD}$ for pedigrees with three disease-affected relatives.

Genetic Relative Risk, κ	Sample Size	$P(\mathcal{A}_{IBD} = \alpha)$							
		α							
		0.1458	0.2083	0.2292	0.25	0.2917	0.3333	0.4167	0.5
$\kappa = 1$	40	0.0000	0.1750	0.0000	0.0500	0.1250	0.2750	0.1000	0.2750
$\kappa = 10$	143	0.0140	0.0490	0.0070	0.0280	0.0070	0.1958	0.2238	0.4755
$\kappa = 20$	191	0.0000	0.0209	0.0000	0.0314	0.0471	0.1518	0.2042	0.5445

3 Negative Control for Anticipation: Age at Death

As discussed in the main text, in section *Results: Anticipation*, it is possible to use the ages of death in unaffected relatives as a negative control to gain insight into ascertainment bias that contributes to apparent anticipation signals in age of onset [1]. In this context, an individual's generation number is relative to the eldest pedigree founder. That is, the two eldest founders will have generation number one, their offspring generation number two, etc. Figure 1 displays box plots of age of death for three genetic-relative-risk groups: $\kappa = 1$, $\kappa = 10$, and $\kappa = 20$. In Figure 1, we see that, within genetic-relative-risk group, the age of death tends to decrease successive generations. This apparent anticipation arises from right truncation in younger generations.

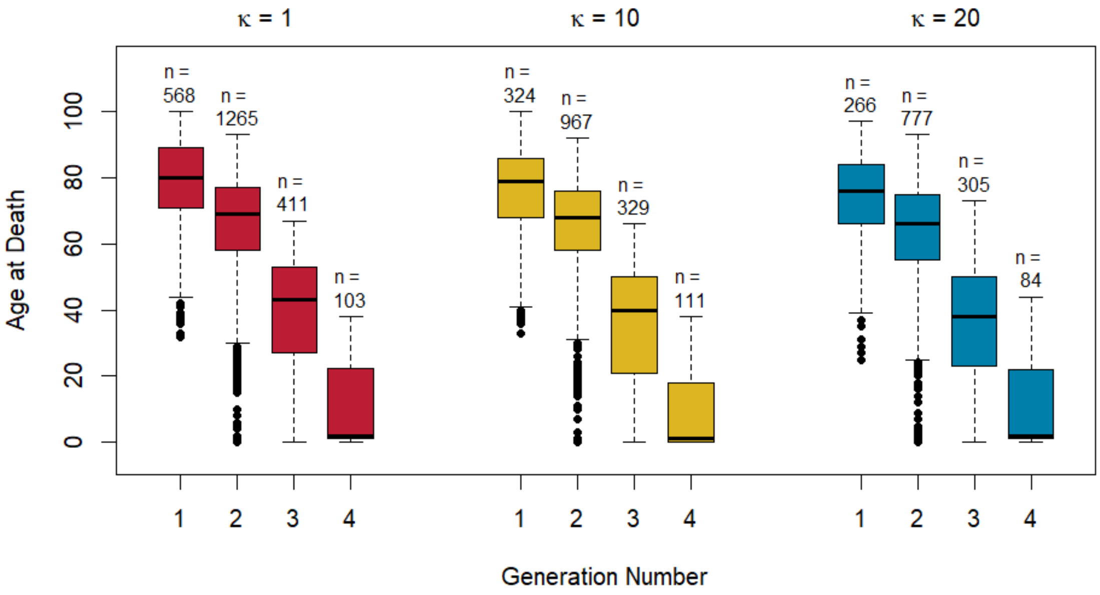

Figure 1: Box plots of age of death in unaffected relatives by generation number grouped by genetic relative-risk of disease, κ. The numbers of observations, n, used to create each box plot are displayed above their respective plots.

4 Effect of Follow Up on Ascertainment Bias

To determine if increasing the time to follow up reduces the effect of the ascertainment bias, we simulated three study samples each containing 500 pedigrees according to the following criteria.

1. Each pedigree was ascertained from the year 2000 to the year 2015.
2. Each pedigree contained at least two relatives affected by lymphoid cancer.
3. The birth year of the founder who introduced the rare variant to the pedigree was distributed uniformly from 1900 to 1980.
4. For each κ considered, the carrier probability, p_c, for all causal variants with genetic-relative risk κ was assumed to be 0.002.
5. Sporadic cases, i.e. affected individuals who did not inherit the rare variant, experienced disease onset according to the baseline, age-specific hazard rate of lymphoid cancer. The population age-specific hazard rates of lymphoid cancer were estimated through the Surveillance, Epidemiology, and End Results (SEER) Program [2, 3].
6. Genetic cases, i.e. affected individuals that inherited the rare variant, experience disease onset at 1, 10, or 20 times the baseline, age-specific hazard rate of lymphoid cancer. That is, for the first sample of 500 pedigrees the genetic relative-risk was set to 1, for the second it was set to 10, and for the third it was set to 20.
7. Since death by lymphoid cancer accounts for a relatively small proportion of all causes of death, the age-specific hazard rate for death in the unaffected population was approximated by that of the general population. Individuals who developed lymphoid cancer experienced death according to the age-specific hazard rate of death in the affected population [2, 5, 6], whereas unaffected individuals experienced death according to the age-specific hazard rate of death in the general population [4].
8. The proband's probabilities for recalling relatives were set to `recall_probs = (1)`; so that pedigrees were fully-ascertained.
9. The stop year of the study was set to 2115.

We restrict attention to pedigree members who were alive at the time of ascertainment. Individuals born after 2015 were not considered. For the three genetic-relative-risk groups considered (1, 10, and 20), we compare the distribution of age of onset by assigned generation number for disease-affected relatives at various follow-up milestones. We consider the following milestones: at the end of the ascertainment period or the 0-year milestone (2015), at the 25-year milestone (2040), at the 50-year milestone (2065), and at the 100-year milestone (2115). Figure 2 displays box plots of the age of onset for the three groups and four milestones considered.

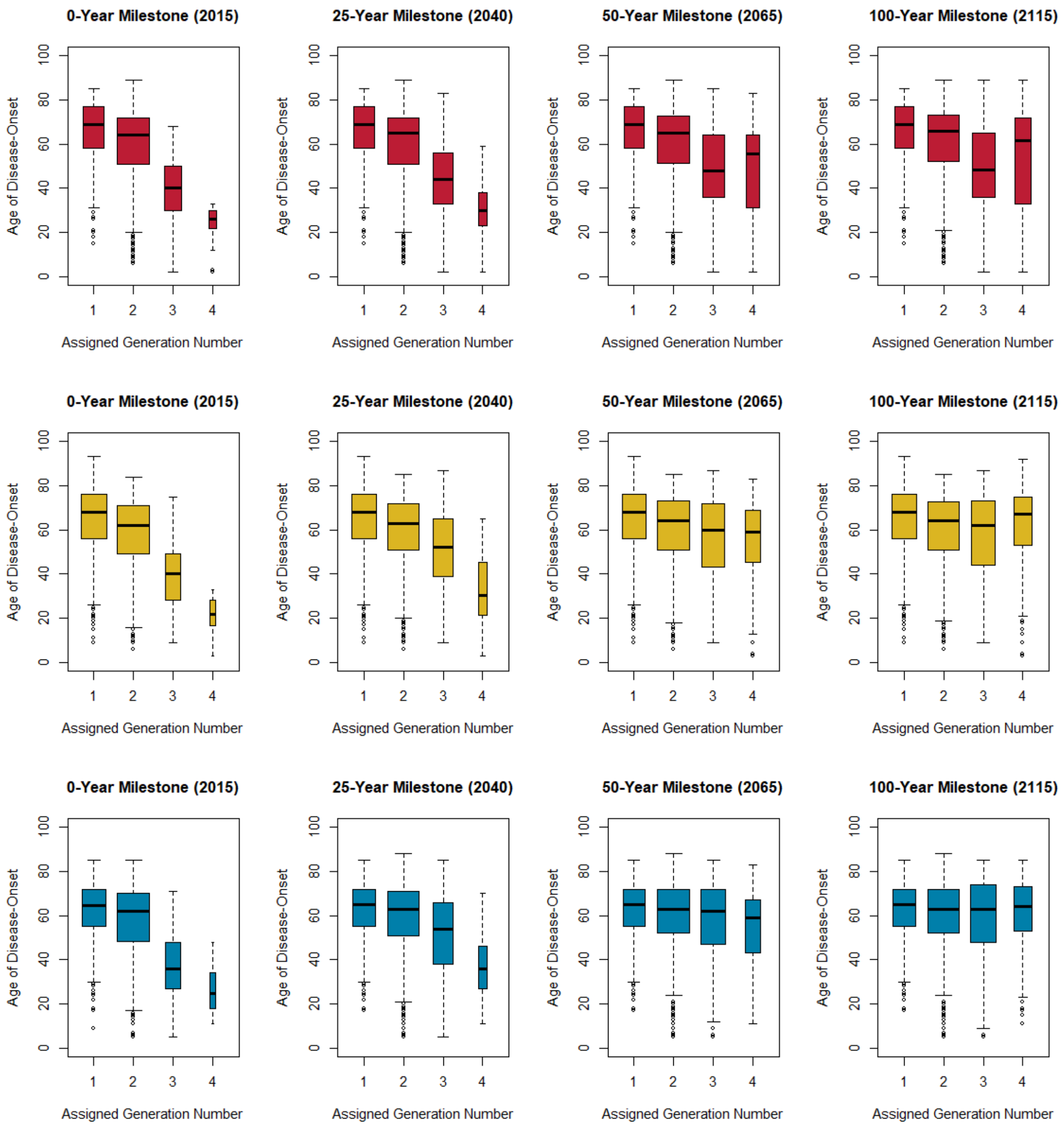

Figure 2: Box plots of age of onset for disease-affected relatives by assigned generation number (see main text) at 0, 25, 50, and 100 years to follow-up for the three relative-risk groups considered. From top to bottom, the first row provides results for the $\kappa = 1$ (fully sporadic) sample, the second row provides results for the $\kappa = 10$ sample, and the third row provides results for the $\kappa = 20$ sample.

From Figure 2 we see that, as the time to follow-up increases and additional relatives experience disease onset, the age of onset for assigned generations three and four shift upward, and appear more like those of generations one and two. Thus increasing the time to follow-up by a considerable amount reduces the effect of ascertainment bias.

5 Effect of Carrier Probability on Proportion of Ascertained Families with Genetic Cases

We illustrate the effect of varying carrier probability on the proportion of ascertained pedigrees that are segregating a genetic variant. To accomplish this, in addition to the one thousand pedigrees considered in *Results: Applications: Proportion of Ascertained Pedigrees Segregating a Causal Variant* we simulated an additional one thousand pedigrees, according to the same settings described in the main text, with carrier probability 0.01 and 0.005. The results of this investigation are displayed in Figure 3.

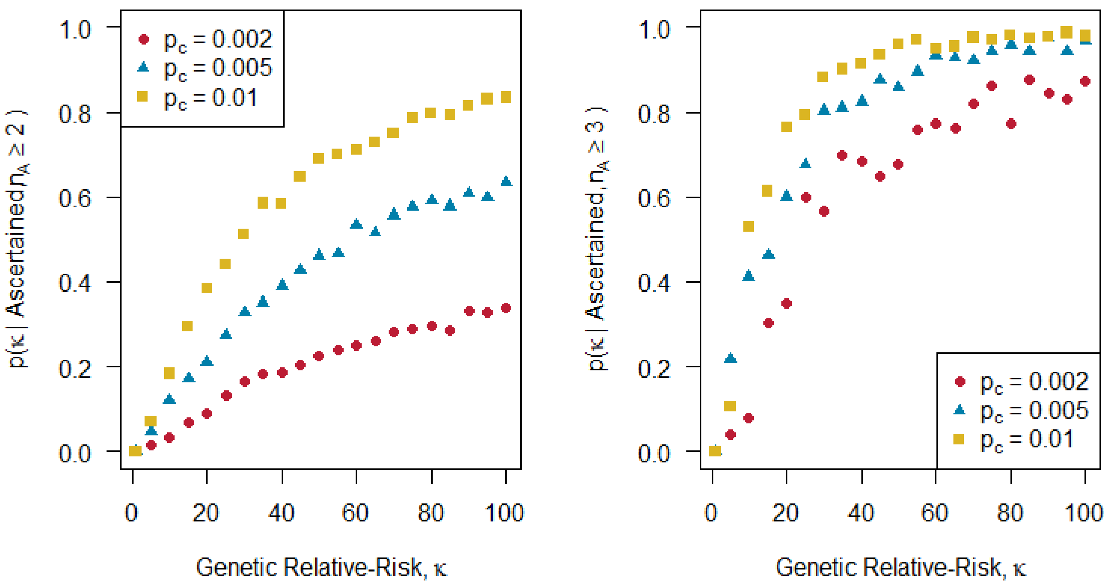

Figure 3: Scatter plots of the probability that a randomly selected pedigree from a sample of ascertained pedigrees is segregating a genetic variant with carrier probability p_c and relative-risk of disease κ against the relative-risk of disease κ. We consider restricting attention to the ascertained pedigrees with n_A or more disease-affected relatives. In the leftmost plot, we consider all one thousand pedigrees ascertained with two or more disease-affected relatives; in the rightmost plot, we consider the subset with three or more disease-affected relatives.

Figure 3 illustrates that as the carrier probability increases the proportion of ascertained pedigrees that segregate a causal variant increases for any genetic relative-risk value considered except when $\kappa = 1$.

Our simulation procedure only allows the starting founder, and not any of the marry-ins, the opportunity to introduce a causal variant. Therefore, as the carrier probability increases our procedure will introduce a causal variant less frequently than would be observed under the assumptions of random mating in the population. As a result, as p_c increases this procedure will underestimate the proportion of ascertained families that are segregating a causal variant.

6 Comparison of Simulated and Observed Age-Specific Fertility Data

We demonstrate that the proposed method to simulate the waiting time to reproduction, described in *Methods: Simulating Life Events: Reproduction*, mimics observed fertility data. We simulated 10,000 lives starting at birth and ending with death, and recorded the ages at which each individual reproduced. From this data we calculated the percentage of first-born births by age group. Table 3 compares the percentage of first-born births by age group in the simulated data with that of the 1993 and 2013 Canadian populations [7].

Table 3: Comparison of percentage of first-born live birth by age group in the Canadian population with the simulated fertility data.

	Percentage of First-Born Live Births by Age Group		
age group	Canadian Population 1993	Canadian Population 2013	Simulated Data
Under 20	11.6	6.0	9.3
20 to 24	25.8	18.0	27.7
25 to 29	35.9	33.3	35.5
30 to 34	20.5	29.9	19.7
35 to 39	5.4	10.7	6.7
40 to 49	0.7	2.2	0.7

References

[1] Minikel, E.V., Zerr, I., Collins, S.J., Ponto, C., Boyd, A., Klug, G., Karch, A., Kenny, J., Collinge, J., Takada, L.T., Forner, S., Fong, J.C., Mead, S., Geschwind, M.D.: Ascertainment Bias Causes False Signal of Anticipation in Genetic Prion Disease. The American Journal of Human Genetics (2014). doi: 10.1016/j.ajhg.2014.09.003

[2] Surveillance Research Program, National Cancer Institute SEER*Stat software, Version 8.3.1.

[3] Surveillance, Epidemiology, and End Results (SEER) Program, SEER*Stat Database: Incidence - SEER 18 Regs Research Data + Hurricane Katrina Impacted Louisiana Cases, Nov 2014 Sub (2000-2012) <Katrina/Rita Population Adjustment> - Linked To County Attributes - Total U.S., 1969-2013 Counties, National Cancer Institute, DCCPS, Surveillance Research Program, Surveillance Systems Branch, released April 2015, based on the November 2014 submission. Available from: http://www.seer.cancer.gov/.

[4] Social Security Administration, Social Security Actuarial Life Tables, Life Tables for the United States Social Security Area 1900-2100. Available from: www.ssa.gov/oact/NOTES/as120/LifeTables_Tbl_6_2000.html.

[5] Surveillance, Epidemiology, and End Results (SEER) Program, SEER*Stat Database: Incidence - SEER 9 Regs Research Data, Nov 2014 Sub (1973-2012) <Katrina/Rita Population Adjustment> - Linked To County Attributes - Total U.S., 1969-2013 Counties, National Cancer Institute, DCCPS, Surveillance Research Program, Surveillance Systems Branch, released April 2015, based on the November 2014 submission. Available from: http://www.seer.cancer.gov/.

[6] Surveillance, Epidemiology, and End Results (SEER) Program, SEER*Stat Database: Incidence-Based Mortality - SEER 9 Regs Research Data, Nov 2014 Sub (1973-2012) <Katrina/Rita Population Adjustment> - Linked To County Attributes - Total U.S., 1969-2013 Counties, National Cancer Institute, DCCPS, Surveillance Research Program, Surveillance Systems Branch, released April 2015, based on the November 2014 submission. Available from: http://www.seer.cancer.gov/.

[7] Statistics Canada. 2016. “Chart 3 First-born live births, by age group of mother, Canada, 1993, 2003, and 2013” (Data table). Health Fact Sheets: Trends in Canadian births, 1993 to 2013. Statistics Canada Catalogue no. 82-625-X. Ottawa, Ontario. https://www150.statcan.gc.ca/n1/pub/82-625-x/2016001/article/14673-eng.htm (accessed September 18, 2018).
